# Positive cytoplasmic UCHL5 tumor expression in gastric cancer is linked to improved prognosis

**DOI:** 10.1371/journal.pone.0193125

**Published:** 2018-02-23

**Authors:** Leena Arpalahti, Alli Laitinen, Jaana Hagström, Harri Mustonen, Arto Kokkola, Camilla Böckelman, Caj Haglund, Carina I. Holmberg

**Affiliations:** 1 Research Programs Unit, Translational Cancer Biology Program, University of Helsinki, Helsinki, Finland; 2 Department of Surgery, University of Helsinki and Helsinki University Hospital, Helsinki, Finland; 3 Department of Pathology, University of Helsinki and HusLab, Helsinki University Hospital, Helsinki, Finland; National Cancer Center, JAPAN

## Abstract

Gastric cancer is the second most common cause of cancer-related mortality worldwide. Accurate prediction of disease progression is difficult, and new biomarkers for clinical use are essential. Recently, we reported that the proteasome-associated deubiquitinating enzyme UCHL5/Uch37 is a new prognostic marker in both rectal cancer and pancreatic ductal adenocarcinoma. Here, we have assessed by immunohistochemistry UCHL5 tumor expression in gastric cancer. The study cohort comprised 650 patients, who underwent surgery in Helsinki University Hospital, Finland, between 1983 and 2009. We investigated the association of cytoplasmic UCHL5 tumor expression to assess clinicopathological parameters and patient survival. Positive cytoplasmic UCHL5 tumor immunoexpression is linked to increased survival of patients with small (<5 cm) tumors (*p* = 0.001), disease stages I-II (*p* = 0.025), and age 66 years or older (*p* = 0.037). UCHL5 is thus a potential marker in gastric cancer with new prognostic relevance.

## Introduction

Gastric cancer is an aggressive disease, with a 5-year survival rate in the Western world of less than 30%, despite improvements in surgical and oncological treatments [1,2]. As the path of the disease varies, and current TNM staging is insufficient in accurately predicting patient survival, new tumor biomarkers for clinical use are vital. We have recently demonstrated that patients with high tumor immunoexpression of the deubiquitinating enzyme UCHL5/Uch37 had better prognosis in colorectal cancer (CRC) and pancreatic ductal adenocarcinoma (PDAC) [3,4].

Modulators of the ubiquitin-proteasome system (UPS) have recently emerged as attractive targets in cancer therapy [5,6]. The majority of controlled protein degradation is mediated by UPS, which is a critical part of maintaining proteostasis. Proteasome substrates are degraded after their polyubiquitination by E1 to E3 enzymes [7]. Important players include also deubiquitinating enzymes (DUBs) removing ubiquitin(s) from target proteins. Approximately 90 DUBs exist in the human genome, around 40 of which have been linked to cancer [8,9]. Three DUBs associate with the proteasome, including UCHL5/Uch37, a cysteine protease of the family of ubiquitin C-terminal hydrolases (UCHs). The DUB activity of UCHL5 is induced by its reversible and evolutionarily conserved binding to the Admr1/Rpn13 subunit of the 26S proteasome [10,11]. In addition, DUB-inactive UCHL5 associates with the NRFKB subunit of the INO80 chromatin remodeling complex, but the physiological significance of this association is unknown [12,13]. The *C*. *elegans* homolog of UCHL5, UBH-4, tissue-specifically modulates proteasome activity, also affecting the health and lifespan of these animals [11]. UCHL5 function is essential, as UCHL5 knockout in mice is embryonically lethal [14].

In cancer and healthy tissues both the expression level and subcellular location of UCHL5 vary greatly [15]. In esophageal squamous cell carcinoma, hepatocellular carcinoma, and epithelial ovarian cancer, high UCHL5 expression correlated with cancer recurrence and reduced patient survival [16–18]. We recently demonstrated that patients with high UCHL5 nuclear positivity in PDAC, and strong positive UCHL5 expression in lymph-node-positive (Dukes C/stage III) rectal cancer, showed a more favorable prognosis [3,4]. Here, we have investigated the potential prognostic role of UCHL5 in gastric cancer.

## Materials and methods

### Patients

Samples for this study came from patients treated for gastric cancer at the Department of Surgery, Helsinki University Hospital, Finland, between 1983 and 2009. The study cohort comprised 650 patients, who underwent total or partial gastrectomy with D1- or D2-lymphadenectomy; 371 (57.1%) of them with curative intent. Their median age at the time of surgery was 66.9 (interquartile range 57.0–75.0), and 335 (51.5%) were women. Median follow-up time was 1.6 (interquartile range 0.6–4.7) years. Clinical data came from patient records, and survival data from the Population Register Centre of Finland; cause of death was provided by Statistics Finland [19,20]. The study was approved by the Surgical Ethics Committee of Helsinki University Hospital (Dnro HUS 226/E6/ 06, extension TMK02 §66 17.4.2013), and the National Supervisory Authority of Welfare and Health gave permission to use the tissue samples without individual consent in this retrospective study (Valvira Dnro 10041/06.01.03.01/2012).

### Preparation of tumor tissue microarrays and immunohistochemistry

The surgical tissue samples provided by the Department of Pathology, Helsinki University Hospital, were formalin-fixed and paraffin-embedded. The patient tissues were de-identified and analyzed anonymously. All histological slides were re-evaluated by an experienced pathologist, who defined and marked areas representing the highest grade of an individual tumor. For the preparation of tissue microarray blocks (TMA), representative areas of tumor specimens were defined and marked on hematoxylin- and eosin-stained slides. A semiautomatic tissue microarrayer (Tissue Arrayer 1, Beecher Instruments Inc., Silver Spring, MD, USA) was used to take three 0.6 mm (1983–1999) or four 1.0 mm (2000–2009) cores from each tumor block, which were then cut into 4-μm sections. Deparaffinization of the slides was done in xylene, and rehydration in a gradually decreasing concentration of ethanol in distilled water. Antigen retrieval was done with a PreTreatment module (Lab Vision Corp., Fremont, CA, USA) in Tris-HCl (pH 8.5) buffer for 20 min. at 98°C. Autostainer 480 (Lab Vision Corp., Fremont, CA, USA) by the Dako REAL EnVision Detection system, Peroxidase/DAB+, Rabbit/Mouse (Dako, Glostrup, Denmark) served for staining of the sections. A rabbit polyclonal anti-UCHL5 antibody (Sigma Aldrich, MO, USA, HPA005908; diluted to 1:800 = 8 μg/ml) was used for one hour at room temperature for tissue incubation. Staining was validated by a similar protocol with a mouse monoclonal anti-UCHL5 antibody (Santa Cruz, TX, USA, sc-271002; diluted to 1:1000) in a subset of 98 samples.

### Evaluation of stainings

UCHL5 cytoplasmic staining was scored according to its intensity as either negative (0), weak positive (1), moderate positive (2), or strong positive (3). The stainings were evaluated first by one investigator (A.L. or L.A.), and then by J.H., independently of any knowledge of clinical data or patient outcome. Possible disagreements with sample scores were discussed until unanimity. Representative images for each staining intensity were chosen at random and image brightness was adjusted equally for all images by Adobe Photoshop.

### Statistical analysis

UCHL5 immunoexpression was dichotomized into either negative (score 0) or positive (scores 1–3), and the maximum score for each sample served for statistical analysis. Fisher’s exact test and linear-by-linear association test served to evaluate any association between UCHL5 expression and clinicopathological variables. Cancer-specific survival (CSS) was calculated from date of surgery to date of death from gastric cancer or until end of follow-up. The Kaplan-Meier method was used for survival analysis, and the log rank-test for comparing the different groups. Exact 95% confidence intervals (CI) were calculated for survival rates. As the median age of patients in the first series (operated 1983–1999) was 66, patients were divided into groups: younger than 66, or 66 and over. Uni- and multivariable survival analysis, used the Cox regression proportional hazard model, and the multivariable model was adjusted for age, gender, stage, and tumor size (≥ 5 cm vs. <5 cm). The Cox model assumption of constant hazard ratios over time was tested. A time-dependent covariate was included separately for each testable variable at a time. All variables fulfilled the assumption, except in one multivariable model (see Results). Interaction terms were considered. A *p* value less than 0.05 was considered significant, with all tests two-sided. Statistical analyses were performed with SPSS version 24.0 (IBM SPSS Statistics, version 24.0; SPSS, Inc., Chicago, IL, USA, an IBM Company), and with SAS version 9.4 (SAS Institute Inc., Cary, NC, USA).

## Results

### Immunohistochemical staining and association of UCHL5 immunoexpression with clinicopathological parameters

**Fig 1:**
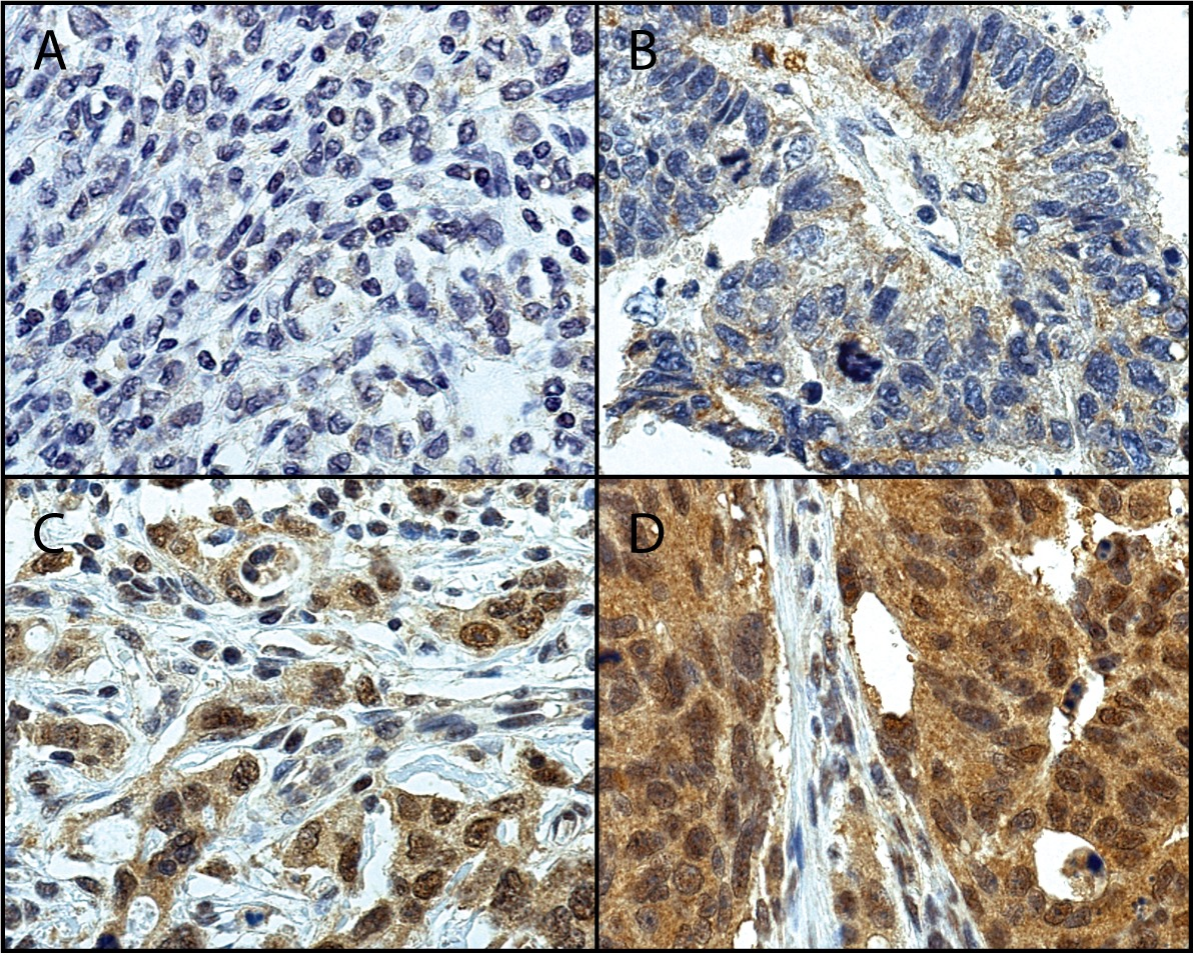
Representative images of UCHL5 staining in gastric cancer. Tumors with A) negative (0), B) weak positive (1), C) moderate positive (2), and D) strong positive (3) staining. Original magnification 40x.

UCHL5 immunoexpression was evaluable in 490 (75.4%) of the tumor specimens. A cytoplasmic and nuclear UCHL5 staining pattern was observable when present throughout the tumor tissue, and was of uniform intensity. Cytoplasmic UCHL5 expression was negative in 111 (22.7%), weak in 217 (44.3%), moderate in 119 (24.3%), and strongly positive in 43 (8.8%) samples. UCHL5-positive cytoplasmic immunoexpression occurred in 379 (77.3%) samples. Due to overlapping cytoplasmic and nuclear staining in a large number of samples, separate evaluation of nuclear staining was not possible. Staining of a subset of samples with a different anti-UCHL5 antibody displayed similar UCHL5 immunoexpression pattern (data not shown). Tumor-adjacent normal-appearing tissue exhibited, for the most part, low staining. Representative images for all intensities (0–3) are depicted in Fig 1. Positive UCHL5 immunoexpression associated with intestinal cancer type (*p* = 0.004), but not with any of the other clinicopathological variables (Table 1).

**Table 1:** Association of UCHL5 with clinicopathological variables in gastric cancer patients.

	UCHL5		
	Negative	Positive	
n (%)	111 (22.7)	379 (77.3)	*p* value
**Age, years**			
<66	51 (22.0)	181 (78.0)	0.747
≥66	60 (23.3)	198 (76.7)	
**Gender**			
Male	54 (22.3)	188 (77.7)	0.914
Female	57 (23.0)	191 (77.0)	
**TNM stage**			
IA-IB	21 (20.8)	80 (79.2)	0.619
II	19 (21.3)	70 (78.7)	
IIIA-IIIC	41 (24.6)	126 (75.4)	
IV	30 (22.7)	102 (77.3)	
Unavailable data		1	
**pT-classification**			
pT1	16 (22.5)	55 (77.5)	0.560
pT2	16 (21.9)	57 (78.1)	
pT3	39 (20.4)	152 (79.6)	
pT4	40 (25.8)	115 (74.2)	
**pN-classification**			
pN0	35 (20.5)	136 (79.5)	0.567
pN+	71 (22.9)	239 (77.1)	
Unavailable data	5	4	
**pM-classification**			
pM0	83 (22.9)	279 (77.1)	0.902
pM1	28 (21.9)	100 (78.1)	
**Laurén classification**			
Intestinal	34 (16.3)	175 (83.7)	0.004
Diffuse	75 (27.5)	198 (72.5)	
Unavailable data	2	6	
**Tumor size, cm**			
<5	46 (26.0)	131 (74.0)	0.173
≥5	61 (20.3)	240 (79.7)	
Unavailable data	4	8	

Abbreviations: UCHL5 = ubiquitin C-terminal hydrolase L5; Unavailable data: number of patients lacking the subgroup characteristic.

### Survival analysis

**Fig 2:**
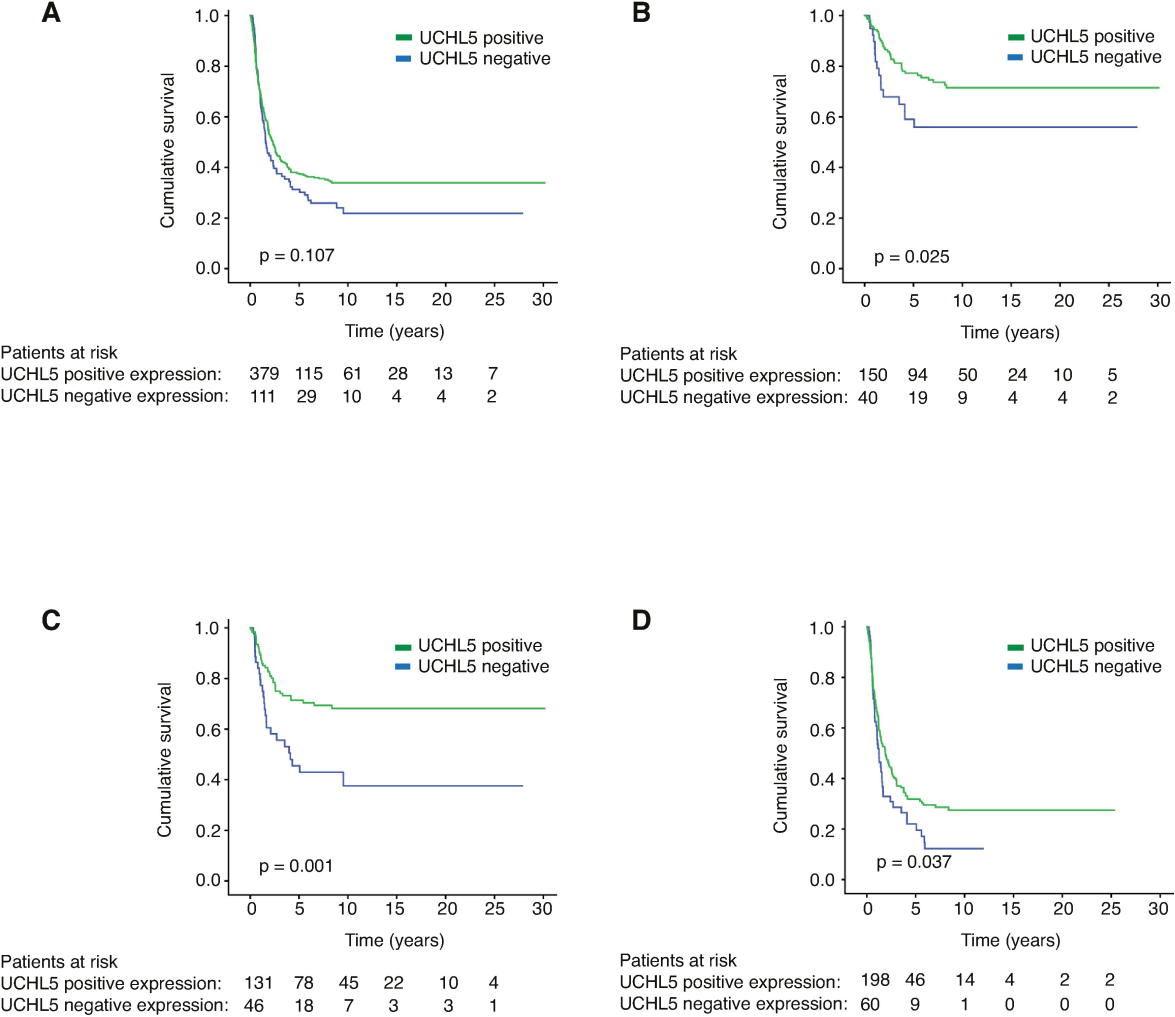
Gastric cancer-specific survival presented by the Kaplan-Meier method. Negative (no staining) versus positive (weak, moderate, and strong positive staining) UCHL5 immunoexpression was compared in A) the whole patient cohort, B) patients with stages I-II of the disease, C) patients with small (<5 cm) tumor size, and D) patients 66 or older. The log rank-test was used for calculating the *p* value.

Positive UCHL5 immunoexpression (staining scores 1–3) was compared to negative immunoexpression (staining score 0) in the entire patient cohort (Fig 2A). More detailed analysis revealed that patients with positive UCHL5 expression exhibited better survival than those with negative expression in the subgroups of patients with stages I-II of the disease (*p* = 0.025, Fig 2B), those with small (<5 cm) tumor size (*p* = 0.001, Fig 2C), and in patients age 66 or older (*p* = 0.037, Fig 2D).

The 5-year cancer-specific survival (CSS) of stages I-II gastric cancer was 77.2% (95% CI 69.2–83.4) in patients with positive UCHL5 immunoexpression, compared to 59.0% (95% CI 41.3–73.0) in patients with negative UCHL5 immunoexpression. In the subgroup of patients with small (<5 cm) tumors, 5-year CSS was 71.4% (95% CI 62.3–78.6) for UCHL5-positive patients and 45.5% (95% CI 30.0–59.7) for those UCHL5-negative. Patients 66 or older with positive UCHL5 immunoexpression displayed a 5-year CSS of 31.8% (95% CI 24.9–38.9), compared to 22.0% (95% CI 11.8–34.2) for negative UCHL5 immunoexpression (Table 2).

**Table 2:** Kaplan-Meier analysis for cancer-specific survival stratified by subgroups of gastric cancer patients.

	5-year cumulative survival % (95% CI)			
	All patients	UCHL5 negative	UCHL5 positive	*p* value
**UCHL5**	36.2 (31.7–40.7)	31.3 (22.5–40.4)	37.7 (32.5–42.8)	0.107
**Age, years**				
<66	42.9 (36.3–49.4)	41.2 (27.4–54.5)	43.4 (35.9–50.7)	0.789
≥66	29.5 (23.6–35.6)	22.0 (11.8–34.2)	31.8 (24.9–38.9)	0.037
**Gender**				
Male	35.1 (28.7–41.6)	30.7 (18.4–43.9)	36.4 (29.0–43.8)	0.300
Female	37.1 (30.9–43.3)	31.9 (20.0–44.5)	38.7 (31.6–45.8)	0.221
**Laurén classification**				
Intestinal	37.7 (30.7–44.7)	30.4 (15.1–47.3)	39.1 (31.4–46.8)	0.193
Diffuse	34.7 (28.9–40.6)	31.1 (20.7–42.0)	36.1 (29.2–43.0)	0.335
**TNM stage**				
IA-IB	86.8 (77.8–92.3)	77.5 (50.4–91.0)	89.1 (79.3–94.4)	0.341
IIA-IIB	58.1 (46.5–68.0)	39.5 (17.9–60.5)	63.2 (49.9–73.9)	0.027
IIIA-IIIC	18.7 (12.9–25.3)	27.2 (14.3–41.9)	15.8 (9.8–23.2)	0.529
IV	4.2 (1.6–8.9)	-	5.5 (2.0–11.5)	0.437
**pT-classification**				
pT1	91.0 (81.0–95.9)	86.7 (56.4–96.5)	92.3 (80.7–97.0)	0.282
pT2	64.7 (51.7–75.1)	45.5 (19.6–68.3)	70.3 (55.5–81.0)	0.009
pT3	23.9 (17.8–30.5)	25.2 (12.5–40.0)	23.6 (16.8–31.0)	0.644
pT4	12.0 (7.2–18.1)	9.2 (2.5–21.6)	12.9 (7.2–20.3)	0.364
**pN-classification**				
pN0	66.4 (58.4–73.2)	53.5 (34.9–68.9)	69.7 (60.9–77.0)	0.159
pN+	20.2 (15.7–25.2)	22.6 (13.3–33.4)	19.5 (14.4–25.1)	0.783
**Tumor size, cm**				
<5	64.6 (56.6–71.5)	45.5 (30.0–59.7)	71.4 (62.3–78.6)	0.001
≥5	19.7 (15.2–24.7)	19.0 (10.0–30.3)	19.9 (14.8–25.6)	0.479

Abbreviations: UCHL5 = ubiquitin C-terminal hydrolase L5, CI = confidence interval. Log rank-test is calculated for the entire follow up period (maximum 30 years).

In univariable Cox-regression analysis, positive UCHL5 expression was not a significant prognostic factor (HR = 0.81; 95% CI 0.63–1.05; *p* = 0.108, Table 3). UCHL5 expression did not fulfill the Cox assumption of proportional hazard ratios over time with all patients included for a multivariable model. However, positive UCHL5 expression was a significant prognostic factor in both uni- and multivariable analysis in subgroups of patients with disease stages I-II (*p* = 0.028 and *p* = 0.001, respectively; S1 Table), and in patients with small (<5 cm) tumor size (*p* = 0.001 and *p* <0.001, respectively; S2 Table).

**Table 3:** Cox regression analysis for cancer-specific survival in gastric cancer.

	Univariable survival analysis		
Variable	Hazard ratio	95% CI	*p* value
**Age, years**			
<66	1.00		
≥66	1.48	1.18–1.86	0.001
**Gender**			
Male	1.00		
Female	0.98	0.79–1.23	0.893
**TNM stage**			
IA-IB	1.00		
IIA-IIB	4.12	2.24–7.60	<0.001
IIIA-IIIC	11.18	6.42–19.48	<0.001
IV	32.28	18.63–57.82	<0.001
**pT classification**			
pT1	1.00		
pT2	3.82	1.73–8.40	0.001
pT3	12.50	6.12–25.55	<0.001
pT4	19.21	9.37–39.42	<0.001
**pN classification**			
pN0	1.00		
pN+	4.10	3.06–5.48	<0.001
**pM classification**			
pM0	1.00		
pM1	5.54	4.33–7.10	<0.001
**Laurén classification**			
Intestinal	1.00		
Diffuse	1.10	0.87–1.38	0.438
**Tumor size, cm**			
<5	1.00		
≥5	3.80	2.86–5.05	<0.001
**UCHL5**			
Negative	1.00		
Positive	0.81	0.63–1.05	0.108

Abbreviations: UCHL5 = ubiquitin C-terminal hydrolase L5, CI = confidence interval.

## Discussion

Here, we demonstrate, to our knowledge for the first time, that in certain subgroups of gastric cancer patients, UCHL5 immunoexpression in tumor tissue is linked to increased survival. In stages I-II of the disease, small (<5 cm) tumor size, or age 66 or older, UCHL5 positivity was linked to better prognosis, when compared to patients with negative tumor immunoexpression.

Most gastric cancer samples, according to the Human Protein Atlas, display predominantly moderate to low UCHL5 immunoreactivity [15]. Similarly, moderate to low expression comprised approximately 69% of our cases. Previously, high UCHL5 expression has been linked to poor prognosis or cancer recurrence in esophageal squamous cell carcinoma, epithelial ovarian cancer, and in hepatocellular carcinoma [16–18]. Contrary to these findings, we here demonstrate that positive UCHL5 immunoexpression is a predictor for better survival in certain subgroups of gastric cancer patients. We have recently shown corresponding results in PDAC, where positive nuclear or high cytoplasmic UCHL5 expression predicts better survival [4]. Additionally, patients with high cytoplasmic UCHL5 immunoreactivity had increased survival in lymph node-positive (Dukes C/stage III) rectal cancer [3].

Proteasome-associated UCHL5 is a negative regulator of proteasome activity, as established in both human cancer cell-lines and the nematode *C*. *elegans* [11,12,21]. It is possible that excess UCHL5 expression substantially impairs proteasome activity, causing abnormal accumulation of proteasomal substrates, and harming tumor cells. Interestingly, patients with high UCHL5 expression have better prognosis in later stages of both pancreatic and rectal cancer [3,4], whereas a similar survival benefit was linked to lower stages (stages I-II) of gastric cancer. This may reflect the tissue-specificity that UCHL5 displays both in its expression and in its role in normal cell functions [11]. A decline in proteostasis is one of the hallmarks of aging [22]. For elderly patients with reduced proteostasis capacity, high UCHL5 levels may further inhibit proteasome activity, thereby promoting apoptosis in cancer cells. Alternatively, the longer survival of UCHL5 may be proteasome independent, and conveyed via chromatin remodeling by the UCHL5 interacting partner INO80 [12,13]. However, the strong cytoplasmic expression pattern of UCHL5 does not support a predominantly chromatin remodeling-dependent effect. Further studies are important to discern UCHL5 molecular mechanisms in normal healthy tissues, as well as in different disease conditions.

Today, proteasome inhibitors are in routine clinical use for treatment of such diseases as multiple myeloma [23,24] and mantle cell lymphoma [25], albeit with dose-dependent toxicities, a limited therapeutic window, and substantial drug resistance [26]. Thus, there exists growing interest in therapeutically targeting other components of the UPS, including different DUBs [5,6]. Concurrent pharmacological inhibition of the proteasome-associated DUBs USP14 and UCHL5 reduces proliferation [5,27], and increases survival in multiple myeloma xenograft models [28]. Modulating proteasome activity by targeting one or several of the proteasome-associated DUBs may provide an effective therapeutic option.

Despite recent advances in surgical and oncological therapies, the general prognosis in gastric cancer is poor. Choosing the correct treatment options for individual patients, both before and after possible surgery, may have far-reaching consequences. However, still only a few prognostic markers are in clinical use. The specificity and sensitivity of currently available markers are insufficient for use in diagnosis of gastric cancer. Thus far, reports on CEA or CA 19–9 markers suggest that they may be beneficial in the detection of recurrence, and they are measured in clinical practice [29]. Though the patient number per subgroup in this patient cohort is relatively small, statistical significance and considerable differences in survival emerged. It is therefore conceivable that UCHL5 could have relevant prognostic potential, especially in the context of more extensive patient cohorts.

Among the strengths of this study is a large patient cohort, with long clinical follow-up and survival data. It is important to note, however, that with this TMA method, only a small subsection of the tumor is sampled and scored for immunoexpression. Conversely, though this method may be somewhat crude, it allows processing of a large patient volume in a reasonable time, and by a standardized staining method.

## Supporting information

S1 Table Cox regression analysis for cancer-specific survival of stage I-II gastric cancer patients. (PDF)

S2 Table Cox regression analysis for cancer-specific survival of gastric cancer patients with small (<5 cm) tumors. (PDF)
