# Correction: Positive cytoplasmic UCHL5 tumor expression in gastric cancer is linked to improved prognosis

**DOI:** 10.1371/journal.pone.0197013

**Published:** 2018-05-02

**Authors:** The PLOS ONE Staff

The sixth author, Camilla Böckelman, should be noted as contributing equally to this work with the last author, Carina I. Holmberg.

There are errors in the Funding section. The correct funding information is as follows: This study was supported by the Academy of Finland (grants 259797 and 297776), Medicinska Understödsförenigen Liv och Hälsa r.f. (C.I.H.), Sigrid Jusélius Foundation, Finnish Cancer Foundation (C.H), Victoria Foundation (https://victoriastiftelsen.fi/start/), K. Albin Johansson Foundation (L.A.), Finska Läkaresällskapet, the Kurt och Doris Palander Foundation, and the Martti I. Turunen Foundation (A.L.). The funders had no role in study design, data collection and analysis, decision to publish, or preparation of the manuscript.

The publisher apologizes for these errors.
