# Supplementary material for: Positive cytoplasmic UCHL5 tumor expression in gastric cancer is linked to improved prognosis
Source: PLoS One. 2018 Feb 23;13(2):e0193125. doi: 10.1371/journal.pone.0193125 (PMC5825037; doi:10.1371/journal.pone.0193125)
Supplement: S1 Table — (PDF) [file pone.0193125.s001.pdf]

Supplementary Table 1. Cox regression analysis for cancer-specific survival of stage I-II gastric cancer patients.

	Univariable survival analysis			Multivariable survival analysis		
	**Hazard ratio**	**95% CI**	***p* value**	**Hazard ratio**	**95% CI**	***p* value**
**Age, years**						
<66	1.00			1.00		
≥66	3.18	1.72-5.88	<0.001	2.92	1.54-5.56	0.001
**Gender**						
Male	1.00			1.00		
Female	0.76	0.44-1.30	0.315	0.70	0.40-1.22	0.207
**TNM stage**						
IA-IB	1.00			1.00		
IIA-IIB	4.14	2.24-7.64	<0.001	2.71	1.38-5.29	0.004
**pT classification**						
pT1	1.00			-		
pT2	3.30	1.28-8.51	0.014	-	-	-
pT3	9.40	3.91-22.60	<0.001	-	-	-
pT4	2.78	0.34-23.13	0.343	-	-	-
**pN classification**						
pN0	1.00			-	-	-
pN+	1.53	0.84-2.78	0.164	-	-	-
**Laurén classification**						
Intestinal	1.00			-		
Diffuse	1.09	0.63-1.89	0.761	-	-	-
**Tumor size, cm**						
<5	1.00			1.00		
≥5	3.49	1.99-6.11	<0.001	2.42	1.29-4.53	0.006
**UCHL5**						
Negative	1.00			1.00		
Positive	0.52	0.29-0.93	0.028	0.35	0.19-0.65	0.001

Abbreviations: UCHL5 = ubiquitin C-terminal hydrolase L5, CI = confidence interval
