# Supplementary material for: Positive cytoplasmic UCHL5 tumor expression in gastric cancer is linked to improved prognosis
Source: PLoS One. 2018 Feb 23;13(2):e0193125. doi: 10.1371/journal.pone.0193125 (PMC5825037; doi:10.1371/journal.pone.0193125)
Supplement: S2 Table — (PDF) [file pone.0193125.s002.pdf]

Supplementary Table 2. Cox regression analysis for cancer-specific survival of gastric cancer patients with small (<5 cm) tumors.

	**Univariable survival analysis**			**Multivariable survival analysis**		
	**Hazard ratio**	**95% CI**	***p* value**	**Hazard ratio**	**95% CI**	***p* value**
**Age, years**						
<66	1.00			1.00		
≥66	2.30	1.37-3.87	0.002	3.67	2.11-6.37	<0.001
**Gender**						
Male	1.00			1.00		
Female	0.65	0.39-1.07	0.091	0.70	0.41-1.19	0.189
**TNM stage**						
IA-IB	1.00			1.00		
IIA-IIB	2.53	1.07-5.95	0.034	2.29	0.96-5.42	0.061
IIIA-IIIC	9.25	4.58-18.70	<0.001	10.30	5.06-20.96	<0.001
IV	35.22	15.25-81.33	<0.001	44.70	18.18-109.90	<0.001
**pT classification**						
pT1	1.00			-		
pT2	2.30	0.96-5.48	0.060	-	-	-
pT3	6.61	2.97-14.73	<0.001	-	-	-
pT4	10.87	4.58-25.77	<0.001	-	-	-
**pN classification**						
pN0	1.00			-		
pN+	5.64	3.17-10.03	<0.001	-	-	-
**pM classification**						
pM0	1.00			-		
pM1	12.06	6.29-23.12	<0.001	-	-	-
**Laurén classification**						
Intestinal	1.00			-		
Diffuse	0.78	0.47-1.29	0.326	-	-	-
**UCHL5**						
Negative	1.00			1.00		
Positive	0.43	0.26-0.71	0.001	0.39	0.23-0.66	<0.001

Abbreviations: UCHL5 = ubiquitin C-terminal hydrolase L5, CI = confidence interval
